# Microglial-Targeted nSMase2 Inhibitor Fails to Reduce Tau Propagation in PS19 Mice

**DOI:** 10.3390/pharmaceutics15092364

**Published:** 2023-09-21

**Authors:** Meixiang Huang, Carolyn Tallon, Xiaolei Zhu, Kaitlyn D. J. Huizar, Silvia Picciolini, Ajit G. Thomas, Lukas Tenora, Wathsala Liyanage, Francesca Rodà, Alice Gualerzi, Rangaramanujam M. Kannan, Marzia Bedoni, Rana Rais, Barbara S. Slusher

**Affiliations:** 1 Johns Hopkins Drug Discovery, Johns Hopkins University School of Medicine, Baltimore, MD 21205, USA; mhuang52@jhmi.edu (M.H.); carolynntea@gmail.com (C.T.); xzhu31@jhmi.edu (X.Z.); khuizar1@jhmi.edu (K.D.J.H.); ajit.thomas@jhmi.edu (A.G.T.); ltenora1@jhu.edu (L.T.); rrais2@jhmi.edu (R.R.); 2 Department of Neurology, Johns Hopkins University School of Medicine, Baltimore, MD 21205, USA; 3 Psychiatry and Behavioral Sciences, Johns Hopkins University School of Medicine, Baltimore, MD 21205, USA; 4 IRCCS Fondazione Don Carlo Gnocchi ONLUS, Laboratory of Nanomedicine and Clinical Biophotonics (LABION), 20148 Milan, Italy; spicciolini@dongnocchi.it (S.P.); froda@dongnocchi.it (F.R.); agualerzi@dongnocchi.it (A.G.); mbedoni@dongnocchi.it (M.B.); 5 Center for Nanomedicine, Department of Ophthalmology, Wilmer Eye Institute, Johns Hopkins University School of Medicine, Baltimore, MD 21231, USA; wghmliy1@jhmi.edu (W.L.); krangar1@jhmi.edu (R.M.K.); 6 Clinical and Experimental Medicine PhD Program, University of Modena and Reggio Emilia, 42100 Modena, Italy; 7 Department of Chemical and Biomolecular Engineering, Johns Hopkins University School of Medicine, Baltimore, MD 21205, USA; 8 Pharmacology and Molecular Sciences, Johns Hopkins University School of Medicine, Baltimore, MD 21205, USA; 9 Department of Oncology, Johns Hopkins University School of Medicine, Baltimore, MD 21205, USA

**Keywords:** Alzheimer’s disease, DPTIP, hydroxyl PAMAM dendrimer, D-DPTIP, extracellular vesicles, neutral sphingomyelinase 2, tau

## Abstract

The progression of Alzheimer’s disease (AD) correlates with the propagation of hyperphosphorylated tau (pTau) from the entorhinal cortex to the hippocampus and neocortex. Neutral sphingomyelinase2 (nSMase2) is critical in the biosynthesis of extracellular vesicles (EVs), which play a role in pTau propagation. We recently conjugated DPTIP, a potent nSMase2 inhibitor, to hydroxyl-PAMAM-dendrimer nanoparticles that can improve brain delivery. We showed that dendrimer-conjugated DPTIP (D–DPTIP) robustly inhibited the spread of pTau in an AAV-pTau propagation model. To further evaluate its efficacy, we tested D-DPTIP in the PS19 transgenic mouse model. Unexpectantly, D-DPTIP showed no beneficial effect. To understand this discrepancy, we assessed D-DPTIP’s brain localization. Using immunofluorescence and fluorescence-activated cell-sorting, D-DPTIP was found to be primarily internalized by microglia, where it selectively inhibited microglial nSMase2 activity with no effect on other cell types. Furthermore, D-DPTIP inhibited microglia-derived EV release into plasma without affecting other brain-derived EVs. We hypothesize that microglial targeting allowed D-DPTIP to inhibit tau propagation in the AAV-hTau model, where microglial EVs play a central role in propagation. However, in PS19 mice, where tau propagation is independent of microglial EVs, it had a limited effect. Our findings confirm microglial targeting with hydroxyl-PAMAM dendrimers and highlight the importance of understanding cell-specific mechanisms when designing targeted AD therapies.

## 1. Introduction

In 2018, approximately 50 million people worldwide had Alzheimer’s disease (AD). This figure is expected to triple in 2050 [1]. AD is characterized by the accumulation of amyloid-β (Aβ) and hyperphosphorylated tau (pTau) proteins in the brain. Several clinical trials have focused on reducing Aβ in the brain using γ-secretase [2,3,4,5] or BACE1 inhibitors [6] to inhibit its production or applying anti-Aβ immunotherapy to clear it from the brain [7,8]. However, to date, the effects have been marginal. This has prompted a renewed focus on pTau, the propagation of which in the brain correlates more strongly with AD progression than with Aβ [9,10]. It has been hypothesized that pTau propagation in the brain occurs, in part, via the extracellular vesicles (EVs) [11,12]. Despite our understanding of the mechanisms of pTau propagation, therapeutic methods to halt the spread of tau have been underexplored.

EVs are secreted by eukaryotic cells and carry proteins, RNAs, and lipids. EVs can be synthesized through two endosomal-sorting-complex-required-for-transport (ESCRT) pathways: the ESCRT-dependent pathway and the ESCRT-independent pathway [13,14]. Ceramides play a crucial role in the ESCRT-independent pathway and are generated by the hydrolysis of sphingomyelin by the enzyme neutral sphingomyelinase 2 (nSMase2). Studies from our lab and other studies have illustrated that genetic and pharmacological inhibition of nSMase2 inhibits EV biogenesis and pTau propagation [15,16,17,18]. Despite its promising translational potential, there are no suitable nSMase2 inhibitors for clinical development. Through a high-throughput screening campaign of over 350,000 compounds, our lab identified DPTIP, a highly selective and nM-potent nSMase2 inhibitor [19]. However, DPTIP poor oral pharmacokinetics (PK), modest brain penetration, and rapid clearance, limiting its clinical translation.

To improve its PK properties, we conjugated DPTIP to a hydroxyl-terminated poly(amidoamine) (PAMAM) dendrimer system designed for brain delivery to create dendrimer-DPTIP (D-DPTIP). Dendrimers represent an innovative class of nanoparticles with the potential to transform the treatment of neuroinflammatory disorders [20,21,22]. Our team has been studying hydroxyl-terminated PAMAM dendrimers and their conjugates for many years in both large and small animal models of neurological and ophthalmic disorders [23,24,25,26,27,28,29,30], as well as in recent Phase 2a trials for severe COVID-19 (ClinicalTrials.gov, NCT04458298) [31]. Our hydroxyl PAMAM dendrimers can cross the blood–brain barrier and specifically target activated immune cells [25,26,30]. This allows them to deliver drugs directly to the site of inflammation, where they can have a maximal therapeutic effect.

Previous studies have used the well-characterized murine adeno-associated virus (AAV)-hTau seed-injection model to mimic pTau propagation observed in AD patients [32,33,34,35,36,37]. In these models, tau propagation was shown to be primarily mediated by microglial EVs [16,38,39,40,41]. Using a similar model, we recently demonstrated that D-DPTIP could significantly inhibit microglial nSMase2 activity and robustly block the spread of tau [15]. To further investigate the therapeutic potential of D-DPTIP, we employed the murine PS19 transgenic model of AD. Unexpectedly, we found that D-DPTIP failed to reverse cognitive deficits, mitigate hippocampal volume loss, or alter tau levels in PS19 mice. Through immunofluorescence analyses, fluorescence-activated cell-sorting (FACS) studies and nSMase2 target-engagement assays, we consistently observed that microglia were the primary cell types containing D-DPTIP. Furthermore, we discovered that D-DPTIP selectively suppressed the release of microglia-derived EVs into plasma in PS19 mice, with no effect on EVs from other cell types. We concluded that D-DPTIP selectively targets and inhibits the activity of nSMase2 in microglia. Consequently, in the AAV-hTau seeded model, where tau propagation relies heavily on microglial EVs, D-DPTIP effectively inhibited the spread of tau. However, in the PS19 mouse model, where tau propagation is predominantly mediated by mechanisms independent of microglial nSMase2 activity, D-DPTIP had no effect [42,43,44,45,46].

## 2. Materials and Methods

### 2.1. Animal Studies and D-DPTIP Dosing

All experiments and animal care were carried out in accordance with the Johns Hopkins University Animal Care and Use Committee guidelines. Animals were housed in a 14 h light/10 h dark cycle until 4 weeks prior to behavioral testing. D-DPTIP was synthesized and conjugated as described previously [15]. D-DPTIP treatment started when the animals were 4 months of age, just before they showed hyperactivity [47]. D-DPTIP was administered intraperitoneally at a 10 mg/kg DPTIP equivalent dose dissolved in 1X phosphate buffer saline (PBS). Vehicle control consisted of administering 1X PBS at 1 mL/kg. Doses were given three times per week. Body weights were measured and recorded weekly throughout the entire treatment period. An equal number of male and female mice were enrolled in each study.

### 2.2. Magnetic Resonance Imaging (MRI)

One week prior to sacrifice, we conducted MRI scans on mice (WT N = 4; PS19 + Vehicle N = 4; PS19 + D-DPTIP N = 4). The MRI scans were performed using a 9.4T spectrometer (Bruker BioSpin Corp., Billerica, MA, USA) with 30 mm diameter volume coil placing around the mouse head. Continuous 2% isoflurane was used for anesthesia throughout the scanning. T2-weighted images were acquired utilizing a rapid-acquisition-with-relaxation-enhancement (RARE) sequence. The following parameters were used: FOV 20 mm × 20 mm, matrix size 200 × 200, 35 slices with a thickness of 0.5 mm, rare factor 8, number of acquisitions 6, TE 30 ms, and TR 3425 ms. Voxel of the hippocampus, ventricle, and total brain were measured using a “medical image labeler” in MATLAB (R2022b Update 4) software.

### 2.3. Behavioral Testing

Four weeks before behavior tests were recorded, the mice were transferred to a reverse light cycle room (12 h dark/12 h light) to acclimate to this cycle. Behavioral studies were performed on mice of WT (N = 18) and PS19 (N = 23). Following an 18-week treatment period, all subjects underwent the Y-Maze spatial recognition test, the novel object recognition test (NORT), and the rotarod test sequentially, with a 3-day interval between each test. For habituation the mice were placed in the behavioral testing room at least 30 min before the start of a test. All tests were conducted by the same experimenters. Between the testing of mice, arenas and objects were cleansed with VIMOBA to prevent odor cues. If behavioral testing occurred on the same day as dosing, the mice received their dose after finishing the behavioral tests.

#### 2.3.1. Y-Maze Spatial Recognition

Y-Maze spatial recognition was utilized to assess novel area preference and spatial memory function. The test used a Y-shaped maze with three arms of equal length (15 inches each) that diverged at equal angles, in tandem. A protocol reported by Sarver et al. [48] was utilized with slight modifications, utilizing AnyMaze tracking software version 7.20 (Stoelting, Wood Dale, IL, USA) to record training (T1) and testing (T2). During both trials, mice were placed in arm C with their snouts oriented toward the central zone. In T1, the novel arm—arm B—was blocked off and the mice were allowed to explore the two familiar arms (A and C) for 10 min. T2 started after a resting period of 3 h. During T2, the novel arm was opened and the mice were allowed to explore the entire maze freely for 5 min. The length of time, in seconds(s), that the mice explored the novel arm was compared to the length of time(s) that the mice explored the familiar arms.

#### 2.3.2. Novel Object Recognition Test (NORT)

NORT was conducted to evaluate memory function and object recognition, as previously reported, in identical square arenas (20 × 20 cm) [49]. Tests were run on training days (T1) and testing days (T2). During training (T1) two identical, by color and shape, objects (familiar objects) were fixed in place in the upper half of the box, equally distance (5 cm) from the walls. The mice were allowed to explore the arena and the objects for 10 min. On the second day, during testing (T2), the righthand familiar object was replaced with a novel object. The mice were allowed to explore for 5 min. Testing was visually recorded and scored by a blinded scorer. The length of time, in seconds, that the mice spent interacting with each object was recorded for analysis. Total time (TT) was calculated as the sum of time(s) the mice spent interacting with novel and familiar objects during T2. The absolute discrimination measure (AD) was calculated as the difference between the time the mice spent interacting with the novel object and the time the mice spent interacting with the familiar object. The relative discrimination measure (RD) was calculated as AD divided by TT and the recognition preference index (RPI) was calculated as the time the mice spent interacting with the novel object divided by TT.

#### 2.3.3. Rotarod

Motor impairment was assessed via rotarod testing. Three trials were conducted on a 3 cm diameter rod on the Rotamex 5 rotarod (Columbus Instrument, Columbus, OH, USA) with setting of Accel: 0.006, ACC-IN: 005, S-Sp: 4.0, and E-Sp: 40.0. For habituation the mice were placed on the rod for 1 minute before starting. The mice were allowed to run for a maximum of 5 min or until they fell off, whichever occurred first. The mice were allowed to rest for 1 minute before starting the next trial. The maximum time of each of three successful trials was recorded and averaged for later analysis of latency to fall.

### 2.4. Sample Collection

After 20 weeks of treatment, the mice were euthanized by isoflurane (Primal Critical Care, Bethlehem, PA, USA) overdose and their brain tissues were harvested following blood collection for immunofluorescent staining and immunoblotting analysis. For the purposes of immunostaining, the mouse brains were collected after perfusion with ~15 mL of ice-cold 1X PBS followed by ~15 mL of an ice-cold 4% paraformaldehyde (PFA) (#15714-S, Electron Microscopy Sciences, Hatfield, PA, USA) solution. The brains were postfixed in 4% PFA for 24 h at 4 °C. The brains were transferred to 30% sucrose for 5 days at 4 °C. After dehydration, the brains were embedded in a Tissue-Tek optimal cutting temperature compound (OCT), flash frozen, and stored at −80 °C until used for cryminosectioning. Hippocampal tissues of WT (male N = 6; female N = 6) and PS19 (vehicle-treated male N = 6, vehicle-treated female N = 6; D-DPTIP-treated male N = 6, D-DPTIP female N = 6) mice were harvested fresh and stored at −80 °C until use. The blood was collected via cardiac puncture and placed into lithium heparin microtubes (#41.1393.105, Sarstedt, Nümbrecht, Germany). Plasma samples were collected from the blood by centrifugation at 500× *g* for 10 min and stored at −80 °C until bioanalysis.

### 2.5. Immunofluorescence Staining

The OCT-embedded brains were cryosectioned at a 30 μm thickness. Staining was performed according to the previously reported method with a slight modification [50,51]. The sections were permeabilized and blocked with 5% normal goat serum in 1X Tris-buffered saline (TBS) + 0.1% Triton-X (#9002-93-1, Millopore Sigma, St. Louis, MO, USA) for 1 h at room temperature. The sections were then incubated with a primary antibody against pTau Thr181 (1:500, #12885, Cell Signaling Technologies, Danvers, MA, USA) overnight at 4 °C and washed thoroughly with 1X TBS 3 times for 5 min. The sections were incubated in anti-rabbit AlexaFluor 488 (1:1000, #A11034, Invitrogen, Waltham, MA, USA) secondary antibody for 1 h at room temperature before washing with the same procedure. The sections were then stained with AlexaFluor 647 conjugated anti-NeuN (1:500, #D4G40, Cell Signaling Technologies, Danvers, MA, USA) antibody overnight at 4 °C before washing and treating with Hoechst (1:10,000, #33342, Invitrogen, Waltham, MA, USA). The sections were washed for 5 min 3 times with 1X TBS, coverslipped with Prolong Glass Antifade mountant (#P36930, Invitrogen, Waltham, MA, USA) and dried overnight at room temperature before storing at 4 °C. The slides were imaged on an LSM 800 confocal microscope (Zeiss, Jena, Germany).

### 2.6. Immunoblotting

The immunoblotting technique was utilized, as previously described [52]. Hippocampus samples were lysed via RIPA buffer containing 1X PPI (#87785, Thermo Fisher, Waltham, MA, USA) with mechanical homogenization followed by sonication. The bicinchoninic acid solution (BCA) method was used to quantify total protein concentration (#23225, Thermo Fisher, Waltham, MA, USA). Loading samples were prepared by mixing with loading buffer (#NP0007, Invitrogen) and reducing reagent (#NP0009, Invitrogen, Waltham, MA, USA) before denaturing in a metal bath heated to 95 °C for 5 min. Proteins were separated by SDS-polyacrylamide gel electrophoresis, using NuPage 4–12% bis-tris 1.0 mm midi protein gel (#WG1402BOX, Invitrogen, Waltham, MA, USA) and transferred to PVDF membranes (#IB24001, Invitrogen, Waltham, MA, USA) via the iBlot transfer bystem (#IB21001, Invitrogen, Waltham, MA, USA). Total protein staining (#926-11016, Li-Cor, Lincoln, NE, USA) was performed before blocking non-specific binding sites and used for normalization. The membranes were incubated with Blocking solution (#12010020, Bio-Rad, Hercules, CA, USA) for 5 min before incubating with primary antibody tau 46 (1:500, Mouse, #SC-32274, Santa Cruz, Dallas, TX, USA) and pTau (Thr181) (1:500, Rabbit, #D9F4G, Cell Signaling Technology, Danvers, MA, USA) overnight at 4 °C. The membranes were washed with 1XTBS with tween-20 (TBST) 3 times for 5 min and incubated with goat anti-mouse IgG horseradish peroxidase (HRP) secondary (1:1000, #31430, Invitrogen, Waltham, MA, USA) and goat anti-rabbit 488 fluorescent secondary (1:1000, #A11008, Invitrogen, Waltham, MA, USA) for 1 h at room temperature. Utilizing the Bio-Rad ChemiDoc MP imaging system (#12003154, Bio-Rad, Hercules, CA, USA), the membranes were imaged at appropriate wavelengths, depending on the secondary antibody. With horseradish peroxidase (HRP) antibody, the membranes were processed with enhanced chemiluminescence (ECL) substrates (#1705061, Bio-Rad, Hercules, CA, USA) before imaging.

### 2.7. Microglial Isolation

Microglia were separated for target engagement via the microglial isolation beads method. A neural tissue dissociation kit (P) (#130-092-628, Miltenyi Biotec, Gaithersburg, MD, USA) was used, following the manufacturer’s instructions. Briefly, the mice were euthanized via isoflurane overdose and their brains were harvested following perfusion with 15 mL of chilled 1X PBS. One hemibrain was dissected from each brain that was harvested and placed in a 35 mm petri dish, individually, filled with 2 mL of cold Hank’s balanced salt solution (HBSS) without Ca^2+^ and Mg^2+^ (#14170112, Thermo Fisher, Waltham, MA, USA). The tissue was cut into small pieces and transferred into 2 mL low bind tubes before centrifugation at 300× *g* for 2 min at room temperature. The supernatant was aspirated and enzymatically digested in 1.95 mL of enzyme mix 1 and 30 μL of mix 2 (#130-092-628, Miltenyi Biotec, Gaithersburg, MD, USA), separated and followed by an incubation period at 37 °C under slow continuous rotation for 15 and 10 min, respectively. The tissue was dissociated twice and separated by a 10-min incubation period in the same conditions, before cell suspension application via a 70 μm cell strainer over a 50 mL conical tube. Ten mL of HBSS with Ca^2+^ and Mg^2+^ (#24020117, Invitrogen, Waltham, MA, USA) were applied to each cell strainer before centrifugation at 300× *g* at room temperature for 10 min. The supernatant was aspirated. BSA stock solution (1.9 mL) (#130-091-376, Miltenyi Biotec, Gaithersburg, MD, USA) was added to each tube alongside 100 μL of myelin removal beads II (#130-096-733, Miltenyi Biotec, Gaithersburg, MD, USA) to each tube. The tubes were mixed and incubated at 4 °C for 15 min. The cells were washed by adding 8 mL of BSA stock solution per tube and centrifuged at 300× *g* at room temperature for 10 min. The supernatant was aspirated and 2 mL of BSA stock solution was added to each tube. LS Column (#130-042-401, Miltenyi Biotec, Gaithersburg, MD, USA) was prepared in a magnetic field suitable for a MACS Separator by adding 3 mL of buffer through each column. Cell suspension was passed through, along with 4 mL of BSA stock solution, and collected in 50 mL conical tubes. The tubes were centrifuged at 300× *g* at room temperature for 10 min and the supernatant was aspirated. The pellets were resuspended in 960 µL of a rinsing solution (#130-091-222, Miltenyi Biotec, Gaithersburg, MD, USA). Forty µL of CD11b microbeads were added to each tube, mixed well, and centrifuged at 300× *g* at room temperature for 10 min. The supernatant was aspirated and pellets were resuspended in 1 mL of rinsing solution. LS Columns were prepared, as previously described, and cell suspensions were applied, along with 3 mL of rinsing solution that was added 1 mL at a time and collected into a 50 mL tube as microglia. LS Columns were removed from the magnetic field and 5 mL of rinsing solution was added to each column. Immediately, magnetically labelled cells were flushed from each column and collected in separate 50 mL conical tubes as non-microglia. All of the tubes were centrifuged at 300× *g* for 10 min at room temperature and the supernatant was aspirated completely. The pellets were resuspended in 1 mL of rinsing solution and transferred into 2.0 mL low-bind tubes. The tubes were centrifuged at 300× *g* at 4 °C for ten minutes and the pellets were stored in a −80 °C freezer until use.

### 2.8. Fluorescence-Activated Cell Sorting (FACS)

FACS was used for sorting astrocytes, microglia, neurons, and cell samples, as previously published method [53]. We minced the mice brain hemispheres in hibernate A low-fluorescence reagent and utilized mechanical methods to dissociate. The homogenates were passed through a 70 μm cell strainer and subsequently centrifuged at 300× *g* for 10 min. The supernatants were removed and the cell pellets were resuspended and treated with Miltenyi debris removal reagent (#130-109-398, Miltenyi Biotec, Gaithersburg, MD, USA), following the manufacturer’s instructions. The cell pellets were then resuspended in FACS buffer (0.5% bovine serum albumin in PBS) for the staining of the cell surface markers. Then, the cell suspensions were incubated with anti–CD16/CD32 antibody (5 ng/μL, clone 93, #101320, BioLegend, San Diego, CA, USA) for 10 min at 4 °C to block non-specific bindings. Next, the cells were incubated with proper antibodies for 30 min at 4 °C. The following antibodies were used: BV421 rat anti-mouse CD45 (2 ng/μL, clone 30-F11, #103133, BioLegend, San Diego, CA, USA), BV605 rat anti-mouse/human CD11b (2 ng/μL, #101237, Biolegend, San Diego, CA, USA), PE rat anti-mouse ACSA-2 (1:25 dilution, clone IH3-18A3, #130-123-284, Militenyi Biotec, Gaithersburg, MD, USA), AF 488 rat anti-mouse TMEM119 (5 ng/μL, clone V3RT1GOsz, #53-6119-82, Invitrogen, Waltham, MA, USA), and Alexa 488 rat anti-mouse NeuN (5 ng/μL, clone A60, #MAB377X, Millipore sigma, St. Louis, MO, USA). Following the incubation period, the cells were subjected to a washing step and subsequently resuspended in 300 μL of FACS buffer. The gates were confirmed by 7-AAD viability staining solution (#00-6993-50, Invitrogen, Waltham, MA, USA) to distinguish between live and dead cells. CD45+/CD11b+/TMEM119+ microglia, ACSA2+ astrocytes, and NeuN+ neurons were acquired and sorted by Beckman MoFlo XDP Cell Sorter for cell sorting, and we utilized FlowJo for data analysis.

### 2.9. nSMase2 Activity Assay

nSMase2 activities in microglia and non-microglia cells were determined using a modification of previously published methods [19,54]. Briefly, cell lysates were prepared in ice-cold Tris-HCl buffer (0.1 M, pH 7.5) containing protease inhibitors, using a probe sonicator (15 s pulses × 3 with 15 s between pulses). Utilizing the Amplex red enzyme-coupled system, lysate nSMase2 activity measurements were initiated upon the addition of sphingomyelin (SM). SM hydrolysis was followed for 3 h at 37 °C and relative fluorescence units (RFU, Ex/Em 530/590 nm) were measured at the end of the reaction period. Using BioRad’s detergent dompatible protein assay kit, the total protein in the lysates was also determined. Finally, lysate nSMase2 activities were normalized to their respective total protein and data were presented as RFU/mg/h.

### 2.10. EVs Isolation

EVs were isolated from 100 uL of pooled plasma samples of the same experimental group by size-exclusion chromatography using qEVsingle commercial columns (qEVsingle Gen 2, 70 nm, Izon, Christchurch, New Zealand). Freshly filtered PBS was used as eluent, and EV fractions (from 6 to 11) were stored at −20 °C with protease inhibitors, as already performed in our recent work. The total protein content of the EV samples was measured by BCA assay.

### 2.11. Nanoparticle Tracking Analysis (NTA)

Isolated EVs, diluted in filtered PBS, were characterized for their size distribution and concentration by NTA, using NanoSight NS300 Instrument (Malvern Panalytical Ltd., Malvern, UK) equipped with a flow-cell top-plate and a syringe pump to enable analysis in constant flow. Recordings of the EV movements were collected for 60 s, three times for each sample; then, data were analyzed using the NTA software v.3.4.

### 2.12. Surface Plasmon Resonance Imaging (SPRi) Analysis

The SPRi array was prepared by coating the gold surface of a SPRi biochip (Horiba, Scientific SAS, Palaiseau, France) with a self-assembled monolayer of a mixture of thiolated PEG with carboxylic or alcoholic groups, which were then activated with EDC/NHS chemistry, following our previously described protocol [55]. The spotting procedure for the ligand immobilization was performed using the iFOUR dispensing system (M24You). This instrument allowed us to dispense pico/nanoliter drops of ligands onto the surface, thanks to a piezo driven micro-dispenser (PDMD) equipped with a 130 mm long borosilicate glass capillary and a cylindrical piezo ceramic actuator bonded to it. The ligands used for the SPRi analysis were as follows: anti-CD9 (#14-0098, eBioscience), anti-Glast (EAAT1/GLAST-1/SLC1A3 Antibody, #NB100-1869SS, Novus Biologicals LLC, Centennial, CO, USA), anti-PLP1 (#HBM-PLP-50, HansaBioMed, Tallinn, Estonia), IB4 lectin (from Bandeiraea simplicifolia; L3019, Merck KGaA, Darmstadt, Germany), anti-CD171/L1CAM (#14-1719-82, eBioscience), anti-CD11b (#553311, BD Biosciences, San Jose, CA, USA), and anti-CD106 (#MA5-16429, Invitrogen, Waltham, MA, USA) for EVs detection, and anti-IgG (#407402, BioLegend, Inc., San Diego, CA, USA) as a negative control. Four spots per ligand were obtained on the surface of the SPRi biochip. After a night in a humid chamber, the chip was blocked in a solution of ethanolamine (1 M, pH 9) for 30 min, washed with water, and stored at 4 °C until use. The biochip was then loaded in the XelPleX instrument (Horiba Scientific SAS) for the SPRi measurements and calibration was performed by injecting 200 µL of sucrose (3 mg/mL) at a flow rate of 50 µL/min. Experiments were performed using PBS as the running buffer. Three hundred and seventy µL of EVs (from the pool of EV samples from the same experimental group) diluted in PBS were injected into the SPRi flow chamber, with a flow rate of 25 µL/min. The SPRi values at the end of the association phase were collected for each ligand family. These values were related to the relative amount of specific EV families present in the analyzed sample. EzSuite Version 1.4.1.68 (Horiba) and Origin Pro version 2023b (OriginLab, Northampton, MA, USA) software were used for SPRi data analysis. The signal intensity obtained on the anti-IgG spots was subtracted from the signals obtained on the ligands spotted on the same chip. Then, the signals related to the EV injection were normalized to the mean intensity obtained on Anti-CD9 spots, the marker of generic EVs, allowing the comparison of data from different experiments and samples.

### 2.13. Statistical Analysis

Statistical analyses were performed by utilizing GraphPad Prism 10. To assess the normality of the data, the Shapiro–Wilk test was applied with a significance level set at α = 0.05. All of the data passed the normality test. An unpaired Student’s *t*-test was used to compare two groups and one-way or two-way ANOVA was used for more than two groups. All quantitative data were presented as mean ± SEM. The significance levels were indicated as **** *p* < 0.0001, *** *p* < 0.001, ** *p* < 0.01 and * *p* < 0.05.

## 3. Results

### 3.1. D-DPTIP Had No Effect on Recognition or Spatial Memory Deficits in PS19 Mice

**Figure 1:**
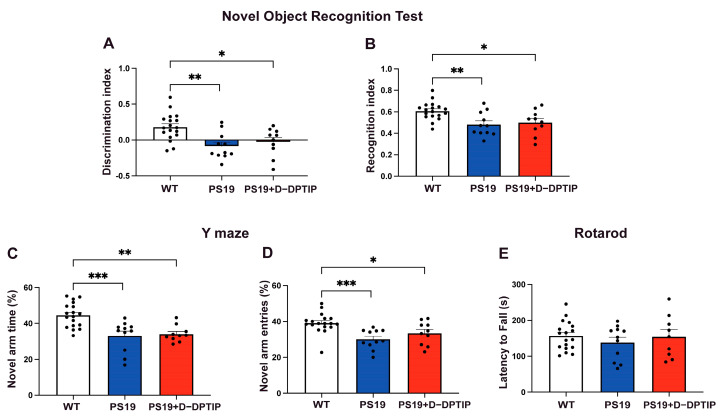
PS19 mice exhibit deficits in recognition and memory that are not improved by D-DPTIP. WT mice with vehicle treatment (N = 18), PS19 with vehicle treatment (N = 11), and PS19 with D-DPTIP treatment (N = 10) mice were used for behavioral tests. The treatment began when the mice were 4 months of age and lasted until they were 10 months old. (**A**) Discrimination index of NORT. Discrimination index = (time spent with novel object − time spent with familiar object)/total time. (**B**) Recognition index of NORT. Recognition index = time spent with novel object/total time. (**C**) Percentage of time spent in the Y-Maze novel arm. (**D**) Percentage of Y-Maze novel arm entries. (**E**) Time to fall in rotarod assessment, measured in seconds. Statistics were performed using one-way ANOVA in (**A**–**E**) with Tukey’s multiple comparisons. Bars represent mean ± SEM. * *p* < 0.05, ** *p* < 0.01, *** *p* < 0.001.

Ten-month-old wild-type (WT) mice, PS19 mice treated with vehicle for 5 months, and PS19 mice treated with D-DPTIP for 5 months were used for behavioral tests. NORT, which was utilized to study recognition memory, showed a significant decrease in the discrimination and recognition indices in the PS19 mice, compared to age-matched WT mice (Figure 1A,B). Treatment of the PS19 mice with D-DPTIP had no effect on NORT. The Y-Maze, utilized to study spatial memory deficits, showed a deficit in the PS19 mice that was unaffected by D-DPTIP treatment (Figure 1C,D). Rotarod was used as a control to ensure that the differences in NORT and Y-Maze were not influenced by motor differences between the groups. No significant differences were observed between the groups (Figure 1E), indicating that motor function did not affect the behavior tests.

### 3.2. D-DPTIP Had No Effect on Hippocampal Atrophy or Ventricular Enlargement in PS19 Mice

**Figure 2:**
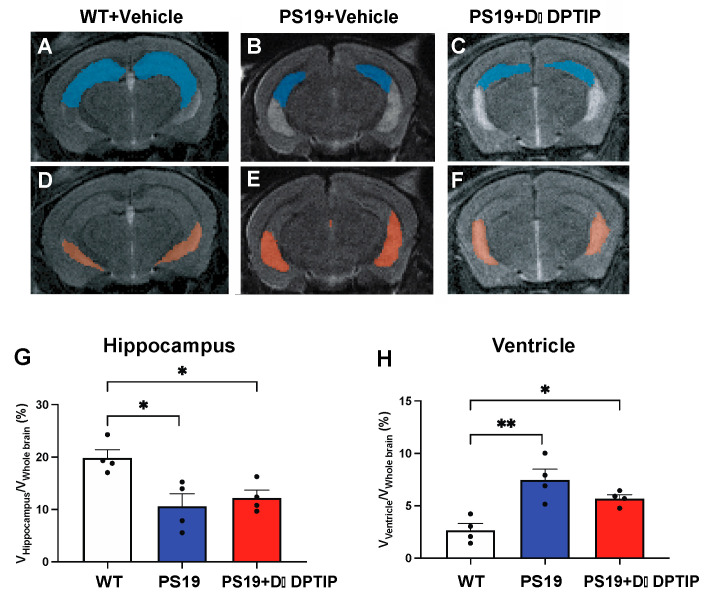
PS19 mice exhibit hippocampal atrophy and enlarged ventricles which are not improved by D-DPTIP. (**A**–**F**) Representative MRI images from WT + vehicle (**A**,**D**), PS19 + Vehicle (**B**,**E**), PS19 + D-DPTIP (**C**,**F**); the hippocampus area was marked as blue (**A**–**C**) and the ventricle area was marked as orange (**D**–**F**). N = 4 mice/group. (**G**) Quantification of hippocampal voxels. (**H**) Quantification of ventricle voxels. Hippocampal and ventricle voxels were normalized by whole-brain voxels. Statistics were performed using one-way ANOVA in (**G**,**H**) with Tukey’s multiple comparisons. Bars represent mean ± SEM. *p* > 0.05 * *p* < 0.05, ** *p* < 0.01.

We employed T2-weighted (T2w) magnetic resonance imaging (MRI) to quantify hippocampal volume and ventricular size in 10-month-old PS19 mice and age-matched WT controls. MATLAB was utilized to calculate the number of voxels corresponding to the hippocampus, the ventricle, and the entire brain. To account for individual variations in brain size, the sizes of the hippocampus and ventricles were normalized by the whole brain volume, expressed as percentages, as previously reported [56]. A significant reduction in hippocampal volume (Figure 2A,B,G) and an increase in ventricular size (Figure 2D,E,H) were observed in the PS19 mice compared to the age-matched WT mice. Treatment with D-DPTIP had no effect on either hippocampal volume (Figure 2B,C,G) or ventricular size (Figure 2E,F,H).

### 3.3. D-DPTIP Had No Effect on Hippocampal Tau or pTau Levels in PS19 Mice

**Figure 3:**
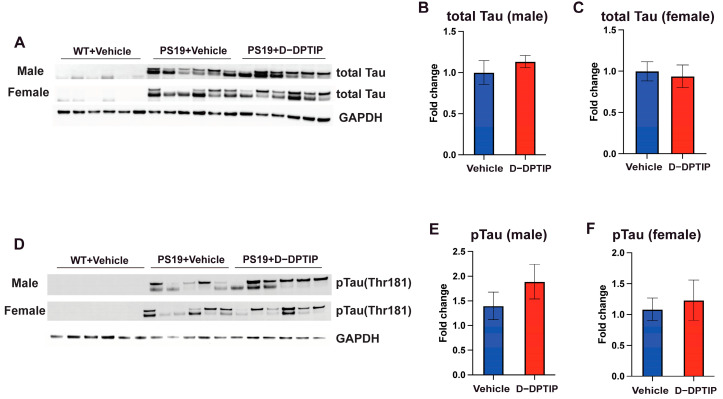
D-DPTIP does not affect tau and pTau expression levels in PS19 mice. (**A**) Representative immunoblotting images from hippocampal tissue of male mice showing total tau (upper blot) and pTau (lower blot). GAPDH was used as a loading control. WT+ vehicle treatment (N = 6), PS19 + vehicle treatment (N = 5), PS19 + D-DPTIP treatment (N = 6) were utilized. (**B**) Quantification of A for total tau normalized by total protein. (**C**) Quantification of A for pTau normalized by total protein. (**D**) Representative immunoblotting images from hippocampal tissue of female mice showing total tau (upper blot) and pTau (lower blot); GAPDH used as a loading control. WT+ vehicle treatment (N = 6), PS19 + vehicle treatment (N = 6), PS19 + D-DPTIP treatment (N = 6) were utilized. (**E**) Quantification of D for total tau normalized by total protein. (**F**) Quantification of D for pTau normalized by total protein. Statistics were performed using unpaired Student’s *t*-tests in (**B**,**C**,**E**,**F**). Bars represent mean ± SEM.

Using immunoblotting to quantify total tau and pTau (Thr181) within the hippocampus, we found that both male and female PS19 mice had large observable bands of total tau and pTau, compared to WT mice that had no discernible bands. D-DPTIP treatment did not affect total tau (Figure 3A–C) or pTau levels (Figure 3D–F) in the PS19 mice.

### 3.4. D-DPTIP Colocalizes with Microglia and Selectively Inhibits Microglial nSMase2 Activity in PS19 Mice

**Figure 4:**
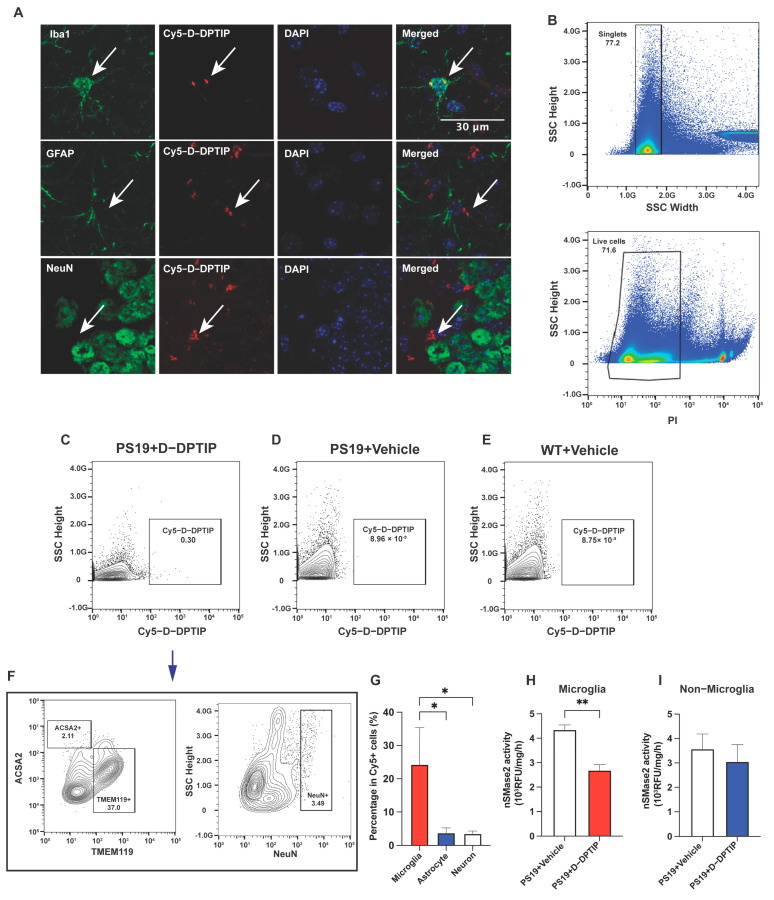
D-DPTIP is preferentially taken up by microglia and selectively inhibits microglial nSMase2 activity. (**A**) Representative hippocampal images showing Cy5–D–DPTIP signal (red) was co-localized with Iba1 (microglia) signal (green), but not with GFAP (astrocyte) or NeuN (neuron) signal (Arrows). (**B**) Single cells were selected by gating singlets (upper panel) and live cells were selected by gating PI negative signals (lower panel). (**C**–**E**) Cy5 positive cells were gated from brain samples of PS19 mice treated with Cy5–D–DPTIP (**C**), PS19 mice treated with vehicle (**D**), and WT mice treated with vehicle (**E**). (**F**) Cy5 positive cells gated from (**C**) were used for gating by ACSA2 (astrocyte) and TMEM119 (microglia) and (NeuN) positive signals. Fluorescence Minus One (FMO) are used for negative controls. (**G**) Quantification of F. Statistics were performed using one-way ANOVA. (**H**,**I**) Quantification of nSMase2 activity assay performed on isolated CD11b- cells (**H**) and CD11b+ cells (**I**). Statistics were performed using unpaired Student’s *t*-tests in (**H**,**I**) and one-way ANOVA in (**G**) with Tukey’s multiple comparisons. Bars represent mean ± SEM. * *p* < 0.05, ** *p* < 0.01. Scale bar is 30 µm.

To elucidate the cellular localization of D-DPTIP within the brain, we employed immunofluorescent staining with Cy5–labelled dendrimer, using techniques we have previously reported [15]. In brief, 24 h after administration of Cy5–labeled D-DPTIP to PS19 mice, the mice were sacrificed and their brains were removed, fixed, sectioned, and stained with antibodies against Iba1 (microglia), GFAP (astrocytes) and NeuN (neurons). We found that Cy5 fluorescent signal predominantly co-localized with Iba1-positive microglia, with minimal co-localization with GFAP-positive astrocytes or NeuN-positive neurons (Figure 4A). To confirm the immunofluorescent findings, we also employed fluorescence-activated cell sorting (FACS), using techniques we have previously described [53]. Twenty-four h after the administration of Cy5–labeled D-DPTIP or PBS (control) to the PS19 mice, we quantified the presence of labelled cells in the brain tissue. We found that 0.30% of all live brain cells from mice receiving Cy5–labeled D-DPTIP exhibited detectable Cy5 signals (Figure 4B,C). In contrast, PS19 and WT mice that received a PBS injection displayed negligible Cy5 signal detection, indicating the success of our gating strategy (Figure 4D,E). Next, we compared the distribution of Cy5–positive cells among specific brain-cell types, specifically microglia (TMEM119+), astrocytes (ACSA2+), and neurons (NeuN+). Among these cell populations, microglia showed an approximately 17.5-fold higher proportion in Cy5+ cells than astrocytes, and a 10.6-fold higher proportion than neurons (Figure 4F,G). These findings support microglia as the primary cell type responsible for the uptake of D-DPTIP. Finally, we conducted ex vivo nSMase2 enzymatic activity assays on microglia-enriched CD11b+ cells (microglial-enriched) and CD11b- (non-microglial) cells isolated from the brains of PS19 mice treated with either D-DPTIP or a vehicle using methods previously described [19,54]. We found that D-DPTIP only inhibited nSMase2 enzymatic activity in the microglial-enriched CD11b+ cells (Figure 4H,I), further highlighting the cellular specificity of D-DPTIP localization in the brain.

### 3.5. D-DPTIP Selectively Reduced the Number of Microglia-Derived EVs in the Plasma of PS19 Mice, with No Effect on EVs from Other Brain-Cell Types

**Figure 5:**
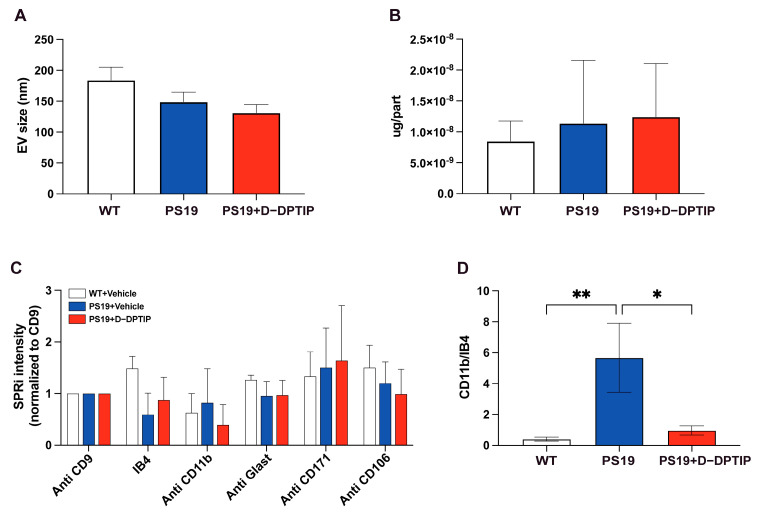
D-DPTIP selectively reduced activated microglia-derived EVs found in PS19 mice plasma. (**A**) EV sizes measured by NTA. (**B**) Protein concentrations per EV particle measured by BCA. (**C**) SPRi analysis intensity of specific brain–cell–type–derived EVs in plasma normalized to CD9 (general EVs). (**D**) CD11b+ (activated microglia) signals normalized by IB4 (total microglia) signals. Statistics were performed using one-way ANOVA in (**A**,**B**,**D**) with Tukey’s multiple comparisons, and two-way ANOVA in C. Bars represent mean ± SEM. * *p* < 0.05, ** *p* < 0.01.

Nanoparticle tracking analysis (NTA) was utilized to assess the impact of D-DPTIP on the size distribution of brain-derived EVs isolated from the plasma of PS19 mice, using methods we previously reported [55]. D-DPTIP had no effect on EV size or EV protein concentration when compared to WT mice and vehicle-treated PS19 mice (Figure 5A,B). To investigate the effects of D-DPTIP on specific brain-derived EVs in plasma, we leveraged surface plasmon resonance imaging (SPRi) technology, as we previously reported [55], which allows for the capturing and profiling of EV populations based on the expression of cell-specific antigens. We prepared an SPRi array by immobilizing antibodies against IB4, CD11b, Glast, PLP1, CD171, and CD106, which specifically captured EVs derived from microglia, activated microglia, astrocytes, oligodendrocytes, neurons, and endothelial cells, respectively. PLP1 data iares not shown because the signal detected was not above background. The SPRi signal intensity analysis revealed no significant changes in EV populations between the PS19 and WT mice or between the D-DPTIP-treated PS19 mice and the vehicle-treated PS19 mice (Figure 5C). However, when the intensity of activated microglia (CD11b+) was normalized to the total microglia signal (IB4+), we found that the PS19 mice displayed an increase in the CD11b+/IB4+ ratio, indicating elevated levels of activated microglia. Notably, D-DPTIP treatment completely normalized this ratio (Figure 5D), again supporting D-DPTIP’s ability to preferentially target activated microglia [15].

## 4. Discussion

In this study, we aimed to investigate the therapeutic potential of D-DPTIP, a potent nSMase2 inhibitor conjugated to a hydroxyl PAMAM dendrimer nanoparticle delivery system, in the PS19 transgenic model of AD. Our previous research utilizing a murine AAV-hTau brain injection and propagation model demonstrated a significant inhibitory effect of D-DPTIP on hTau propagation [15]. Herein, we aimed to extend these findings to evaluate the therapeutic utility of D-DPTIP in the transgenic PS19 mouse model of AD. We evaluated the effect of chronic D-DPTIP administration using multiple cognition and MRI-based endpoints, including discrimination and recognition deficits, spatial memory deficits, hippocampal atrophy, and enlarged brain ventricles. However, despite acceptable tolerance, proven brain penetration, and target engagement of D-DPTIP, we found that it failed to alter any of these pathological features. Additionally, there were no significant changes in total tau or pTau (Thr181) levels following D-DPTIP treatment. Using three independent techniques—immunofluorescent staining, FACS, and nSMase2 activity assay—we showed that D-DPTIP specifically targeted activated microglial cells, with little localization in oligodendrocytes, astrocytes, or neurons. Moreover, D-DPTIP selectively inhibited microglial nSMase2 activity and reversed the increased number of activated microglia-derived EVs in PS19 mice plasma, with no effect on EVs derived from any other brain cell type.

The differential response of D-DPTIP between the AAV-hTau model and the PS19 mice can be attributed to the distinct mechanisms of tau spread in these two models. Recent findings from our lab and from other studies revealed a crucial contribution of microglia to the spread of tau in various AAV-hTau injection and propagation models [16,39,40,41]. Notably, when microglia are depleted using the colony-stimulating factor 1 receptor (CSF1R) inhibitor PLX5622 in an AAV propagation model, tau propagation is significantly halted. More recently, amyloid plaque-associated microglia were shown to exhibit enhanced phagocytosis of tau-containing neurites while hyper-secreting EVs containing pTau, suggesting a link between amyloid plaque deposition, microglia activation, and exacerbation of tau propagation [16,39]. These data in AAV-hTau seeding models suggest essential interactions between microglia and tau propagation.

Given the prominent role of microglial EVs in the propagation of tau in AAV models, in our prior studies, we utilized the hydroxyl PAMAM dendrimer delivery system to target our nSMase2 inhibitors to microglia specifically. We demonstrated that D-DPTIP robustly blocked EV-mediated tau propagation, effectively inhibiting the spread of pTau to the contralateral hippocampus in an AAV-hTau seeding model [15].

Our research team has extensively investigated the application of hydroxyl PAMAM dendrimers in over a dozen nervous system disorders involving six species, including primates [23,24,25,26,27,28,29,30]. We have shown that these hydroxyl PAMAM dendrimers selectively deliver drugs to activated immune cells in the brain [26]. Notably, one of our initial dendrimer conjugates has advanced to clinical development, demonstrating significant therapeutic benefits in preclinical models [24,27], as well as in a recent Phase 2 clinical trial [31].

Utilizing this microglial nSMase2 inhibitor targeting system in the PS19 transgenic mice, however, showed limited effects. Within the PS19 mouse model, there is strong evidence that pathological tau transmits along synaptically connected circuits between neurons. Pathological tau species, such as pTau, undergo misfolding within neurons, forming intracellular tau aggregates that are secreted at the synaptic terminus, in part, through EVs [12,42,43,44,45,46]. Once released, pathological tau aggregates are internalized by neighboring neurons, initiating a cascade of events that contribute to the propagation of tau pathology. Unlike the AAV models described above, when preformed tau fibrils were injected into the locus coeruleus (LC) of the PS19 mouse, tau pathology propagated to brain regions that are anatomically interconnected with LC neurons through either efferent or afferent projections [57], suggesting that the PS19 mouse model exhibits tau propagation primarily through neuronal pathways. Interestingly, the broad inhibition of nSMase2 led to the elimination of tau propagation in PS19 mice [58]. Therefore, addressing this in neurons, rather than just in microglia, may be a key mechanism to produce therapeutic improvements in this model.

Importantly, microglia actively contribute to tau propagation in both AD patients and PS19 mice model [59,60,61,62]. However, the role of microglial nSMase2 is uninvestigated. A recent in vivo study using PET imaging demonstrated the relationship between microglia and tau in 130 individuals spanning the aging and AD spectrum, revealing parallel spatial propagation of microglial activation and tau accumulation along predicted brain circuits [59]. Additionally, investigation of the PS19 mouse model highlighted the significant role of microglial NF-κB signaling in tau spreading and toxicity. Activating NF-κB by tau enhanced microglial-mediated tau pathology, while inhibiting NF-κB activation reduced tau seeding, improved microglial autophagy, and mitigated cognitive deficits associated with tauopathy [61]. Our current findings suggest that while microglia are critical in tau pathology, the specific role of microglial nSMase2-mediated EV biogenesis in PS19 mice may not play a central rsole.

In conclusion, although D-DPTIP exhibits preferential targeting of microglia nSMase2, its limited impact on tau-associated pathologies in PS19 mice highlights the importance of developing strategies that directly address the cell-specific mechanisms underlying tau spread. A comprehensive understanding of these mechanisms is essential for developing effective targeted interventions. Further research in this area is warranted to advance translational considerations and improve therapeutic approaches for attenuating tau-mediated neurodegeneration.

## Data Availability

The data presented in this study are available within the manuscript.
